# A cross sectional study of animal and human colonization with Methicillin-Resistant Staphylococcus aureus (MRSA) in an Aboriginal community

**DOI:** 10.1186/s12889-016-3220-9

**Published:** 2016-07-19

**Authors:** Peter Daley, Janak Bajgai, Carla Penney, Karen Williams, Hugh Whitney, George R. Golding, Scott Weese

**Affiliations:** Department of Medicine and Laboratory Medicine, Memorial University, Room 1 J421 300 Prince Phillip Dr, St. John’s, Newfoundland and Labrador A1B 3V6 Canada; Department of Community Health, Memorial University, St. John’s, Canada; Department of Clinical Epidemiology, Memorial University, St. John’s, Canada; Happy Valley Goose Bay, Labrador, Canada; Department of Public Health, Government of Newfoundland and Labrador, St. John’s, Canada; National Microbiology Laboratory, Winnipeg, Canada; Department of Veterinary Medicine, University of Guelph, Guelph, Canada

**Keywords:** Methicillin-resistant Staphylococcus aureus, Aboriginal, Dog, Colonization

## Abstract

**Background:**

Methicillin-resistant *Staphylococcus aureus* (MRSA) infections are common among humans in Aboriginal communities in Canada, for unknown reasons.

**Methods:**

Cross sectional study of humans and dogs in an Aboriginal community of approximately 1200 persons. Our objectives were to measure community-based prevalence of nasal MRSA colonization among humans, use multivariable logistic regression to analyze risk factors for MRSA colonization, and perform molecular typing of *Staphylococci* isolated to investigate interspecies transmission.

**Results:**

461 humans were approached for consent and 442 provided complete data. 109/442 (24.7 %, 95 % C.I. = 20.7–28.7 %) of humans were colonized with MRSA. 169/442 (38.2 %) of humans had received antibiotics in the last 12 months. Only number of rooms in the house (OR 0.86, *p* = 0.023) and recreational dog use (OR 7.7, *p* = 0.002) were significant risk factors for MRSA colonization. 95/109 (87.1 %) of MRSA strains from humans were of the same spa type (CMRSA10/USA300). 8/157 (5.1 %, 95 % C.I. = 1.7–8.5 %) of dogs were colonized with methicillin-susceptible *S. aureus*, and no dogs were colonized with MRSA.

**Conclusions:**

Human MRSA colonization in this community is very common, and a single clone is predominant, suggesting local transmission. Antibiotic use is also very common. Crowding may partially explain high colonization, but most considered risk factors including animal exposure were not predictive. Very few dogs carried human Staphylococcal strains.

**Electronic supplementary material:**

The online version of this article (doi:10.1186/s12889-016-3220-9) contains supplementary material, which is available to authorized users.

## Background

Methicillin-resistant *Staphylococcus aureus* (MRSA) is a highly virulent bacterial pathogen which is resistant to standard antibiotic therapy. Colonization or infection with this organism is associated with excess morbidity and mortality, prolonged length of hospital stay, and excess cost [1]. Canadian Aboriginal populations seem uniquely affected by community-associated MRSA (CA-MRSA) infection, although little is known about prevalence or risk factors in these settings.

MRSA is an increasing cause of hospital and community-acquired infections, due to selection because of widespread inappropriate antibiotic use in humans [2] and companion animals [3, 4]. MRSA may be present in an asymptomatic state, termed “colonization”, or may cause disease such as skin and soft tissue infections, surgical wound infections, or more invasive infections such as endocarditis, prosthetic hardware infections, or necrotizing pneumonia [5]. Colonization precedes infection in community outbreaks [6].

Regional screening practice for detection of MRSA colonization is variable, meaning population representative colonization rates are not available. The prevalence of MRSA colonization outside of a healthcare setting is not generally available, due to logistical challenges, and representative community subpopulations are generally analyzed, an approach with inherit sampling bias.

MRSA genotyping can describe the molecular epidemiology and transmission of specific types within populations [7]. In Alberta, the proportion of MRSA infections caused by CA-MRSA increased from 19.7 to 36.4 % between 2007 and 2011, and the predominant circulating genotype of CA-MRSA was the CMRSA10/USA300 strain type (22.1 %) [8].

The emergence of MRSA infection among Aboriginal populations has been reported from several countries [9–11]. Surveillance of inpatients in Canada has identified that Aboriginal Canadians are at six times increased risk of CA-MRSA infection as compared to non-Aboriginals [11]. The reason for this difference is unknown, although hypothesized risk factors for CA-MRSA infection include health care exposure, chronic illness, antibiotic use, intravenous drug use, contact with others with risk factors, shared personal hygiene items, athletic teams, skin injury, crowding, skin to skin contact, incarceration, tattoos or piercings, day care, and skin infections [12–14]. Risk factors among Aboriginal communities may not be the same as among non-Aboriginal communities.

In Northern Manitoba, the incidence of MRSA infection increased five-fold between 2003 and 2006 [15]. Surveillance of 1280 clinical isolates in three northern Saskatchewan communities found that 54.1 % of *Staphylococcus aureus* isolates were MRSA, and 98 % of MRSA isolates were CMRSA7/USA400 type [16]. Between January and September of 2010, more than 100 human MRSA infections were reported from Labrador, predominantly originating from two small Aboriginal communities (David Allison, unpublished data). 96.5 % of the 85 strains typed were CMRSA 10. Limited retrospective analysis of clinical information from 141 individuals could not define clinical or epidemiological risk factors.

MRSA may have a zoonotic transmission cycle. MRSA transmission between animals and humans has been demonstrated among domestic pets [17], and veterinary staff and animals [18]. Among animals living in households with one MRSA colonized human, 8.3 % of dogs and 10 % of cats were colonized by the same type of MRSA [19]. A survey of 736 dog owners and 815 dogs demonstrated 23.6 % *Staphylococcus aureus* colonization among owners and 8.8 % colonization among dogs [20]. Although dogs are not commonly colonized by *S. aureus* (14 % of dogs tested in Ontario), they may carry related Staphylococcal strains such as *Staphylococcus pseudintermedius* (46 % of dogs tested in Ontario) [21] or *Staphylococcus sciuri* [22]. The *S. sciuri* genome contains a *mecA* homologue that may be the evolutionary precursor of *mecA* gene found in MRSA [23]. When all coagulase positive *Staphylococci* are included, 74 % of healthy dogs and 88 % of dogs with skin disease are colonized [24].

The objectives of our study were to observe the point prevalence and risk factors for MRSA colonization among humans in an Aboriginal community by attempting to sample the entire consenting population of the community. Secondly, we wanted to examine the genetic relatedness among coagulase positive Staphylococcal strains isolated from humans and dogs for evidence of interspecies transmission. Identified risk factors may be amenable to interventions to interrupt transmission. An educational intervention in Aboriginal communities in Saskatchewan was successful at reducing MRSA infection rate two-fold [25].

## Methods

### Setting

An Aboriginal community of approximately 1200 persons was selected based on the previous investigation. A letter of support was received from the band council before the study began.

### Population sampling

Between October and December 2014, a researcher (student in Masters of Public Health program at Memorial University) (JB) lived near the community. With the assistance of local public health nursing and a community member, the researcher contacted residents through door to door visits, health and vaccination clinics and public announcements. Consenting participants answered a risk factor questionnaire and provided a single nasal swab (both nares). An attempt was made to contact every member of the community.

Dog swab collection was performed by the Chinook Project, a veterinary outreach project funded by the provincial government and based at the Atlantic Veterinary College, already involved in vaccination, deworming and spay and neuter surgery during annual visits to the area. Dog selection was based on the animals the veterinarians were able to access during the study period. Dogs were sampled using a single combined nasal/oral/inguinal fold/anal swab [24] (and from any draining skin wound), from the community in which humans were sampled, and in a second community. During this collection, dog ownership was established.

The swabs were transported in an insulated container in liquid transport medium at room temperature to the Health Sciences microbiology laboratory in St. John’s. Human swabs were applied to a denim blue chromogenic agar (selective for the growth of MRSA) (Oxoid, Nepean, Canada). After overnight aerobic incubation at 37 °C in the dark, a single predominant colony was confirmed with gram stain, catalase and slide coagulase. Human *Staphylococcus aureus* were referred to the National Microbiology Laboratory for *spa* typing and PCR for *mec*A, *fem*A, *nuc* and Panton Valentine leucocidin as previously described [26]. Amplicons were sequenced in both directions, analyzed using BioNumerics v5.1 (Applied Maths, Austin, TX), and submitted to the online Ridom Spa Server and database (http://www.spaserver.ridom.de) for *spa* type designation [27]. Animal swabs were inoculated into tryptic soy broth and incubated aerobically at 37 °C overnight, then inoculated onto mannitol salt agar for overnight incubation. A single predominant *Staphylococcus* was identified using slide and tube coagulase and MALDi-ToF. Coagulase-positive *Staphylococci* were referred to the University of Guelph, where they were identified using species-specific PCR as described by Sasaki et al. [28]. This assay is able to distinguish six Staphylococcal species including *S. aureus*, *S. hyicus*, *S. schleiferi*, *S. intermedius*, *S. pseudintermedius* and *S. delphini* groups A and B.

### Study type or design

The study was a prospective, observational, cross sectional design, designed to represent an entire isolated community.

### Outcomes

Colonization was reported as a percentage among humans or dogs sampled, and risk factors for colonization, based on self-report, were analyzed using single variable logistic regression (SPSS 20.0, IBM). Risk factors included were Gender, Number of people living in house, Number of sinks in house, Source of drinking water, Ethnicity, Admission to hospital, Surgery in 12 months, Indwelling medical device, Hemodialysis, Previous MRSA, Contact with someone with a skin infection, Contact with someone with MRSA, Lived with someone with MRSA, New tattoo or piercing, Chronic skin condition, Healthcare exposure, Correctional facility exposure, Daycare exposure, Contact of person with healthcare exposure, Contact of person with daycare exposure, Contact of person with homeless shelter exposure, Dog in the house, Own a dog, and Feed a dog (see Additional file 1: Appendix).

Variables statistically significant after univariate logistic regression (cutoff *p* > =0.05) were entered in a multivariate logistic regression model. A forward selection method was used to select variables in a stepwise fashion to include a total of five variables following selection.

## Results

**Fig. 1:**
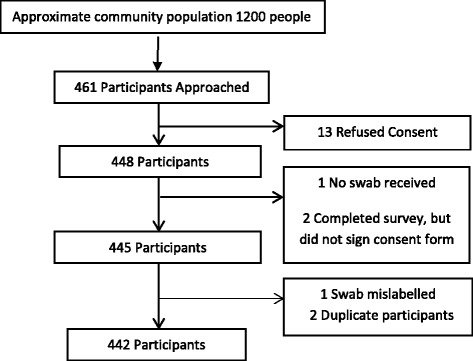
Recruitment of Humans in Aboriginal Community

Four hundred sixty-one humans were approached for consent to participate, from which 442 (95.9 %) provided complete data (Fig. 1). Mean age was 25.7 years (SD 19.8 years), and 45.5 % were female. 169/442 (38.5 %) had received antibiotics in the past 12 months, and 109/442 (24.7 %) were colonized with MRSA (Table 1).

**Table 1:** Demographics and MRSA colonization rate among humans in an Aboriginal community

Age	Mean 25.7 years	SD 19.8 years
Females	201/442 (45.5 %)	
Received antibiotics in 12 months	169/442 (38.2 %)	
MRSA colonized	109/442 (24.7 %)	

Using univariate logistic regression, six risk factors were significantly predictive (Table 2). These risk factors were entered into a multivariate regression and only two were significantly predictive (Table 3): Rooms in house (OR 0.86, 95 % C.I. = 0.75–0.98) and recreational dog use (OR 7.7, 95 % C.I. = 2.1–28.0). The multivariate model had a significance of 0.048, and an R^2^ value of 0.053.

**Table 2:** Risk factors for MRSA colonization of humans, using univariate regression

Risk factor	Positives	OR	*P*=
Rooms in House	Mean 5.3 rooms	0.86	0.036
Antibiotic in 12 months	169/442 (38.5 %)	1.69	0.018
Skin infection in 12 months	119/442 (26.9 %)	1.90	0.006
Incision and drainage in 12 months	24/442 (5.4 %)	2.70	0.020
Contact with person with correctional facility exposure	20/442 (4.5 %)	2.58	0.042
Recreational Dog Use (Hunting, Camping, Walking)	12/442 (2.7 %)	6.35	0.003
Male gender	242/442 (54.8 %)	0.87	0.582
People in house	Mean 2.5 people	0.95	0.116
Sinks in house	Mean 1.1 sinks	0.85	0.293
Additional source of drinking water	201/442 (45.5 %) carried water from outside the house	0.94	0.783
Ethnicity	30/444 (6.8 %) Not Innu	0.91	0.827
Admitted to hospital in 12 months	366/444 (82.8 %) Not admitted	0.76	0.314
Surgery in 12 months	409/444 (92.5 %) No surgery	0.61	0.190
Device in 12 months	395/444 (89.4 %) No device	0.91	0.793
Dialysis in 12 months	441/444 (99.3 %) No dialysis	0.66	0.740
Prior MRSA Colonization	401/444 (90.3 %) No colonization	0.59	0.119
Contact with person with skin infection	256/444 (57.7 %)	1.28	0.268
Contact with person with MRSA colonization or infection	148/444 (33.3 %)	0.85	0.486
Lived with a person with MRSA colonization or infection	136/444 (30.6 %)	0.89	0.635
New tattoo or piercing	32/444 (7.2 %)	0.68	0.400
Chronic skin condition	47/444 (10.6 %)	1.17	0.656
Visited inpatient facility in 12 months	297/444 (66.9 %)	1.10	0.684
Visited correctional facility in 12 months	7/444 (1.6 %)	1.20	0.826
Visited daycare center in 12 months	28/444 (6.3 %)	1.73	0.181
Contact of person exposed in inpatient facility	340/444 (76.6 %)	0.82	0.438
Contact of person exposed to daycare	94/444 (21.1 %)	1.47	0.141
Contact of person exposed to homeless shelter	6/444 (1.4 %)	0.60	0.639
Dog in house	180/444 (40.5 %)	0.91	0.655
Own a dog	144/444 (32.4 %)	1.06	0.815
Feed a dog	178/444 (40.0 %)	0.76	0.219

**Table 3:** Significant risk factors for MRSA colonization of humans, using multivariate regression

Risk factor	OR	*P*=
Rooms in House	0.86	0.023
Recreational Dog Use (Hunting, Camping, Walking)	7.7	0.002

The majority of MRSA isolated from humans contained *spa* types associated with CMRSA10 (95/109, 87.1 %). Of these CMRSA10 isolates, t008 was the predominant *spa* type (86/95, 90.5 %), followed by t121 (5/95, 5.3 %) and t068 (2/95, 2.1 %). Two MRSA isolates were non-typeable by *spa* typing, but determined to fall within the CMRSA10 cluster by pulsed-field gel electrophoresis; these isolates were not related by living in the same household. The remaining 13 MRSA isolates (13/108, 12.0 %) were *spa* type t160, which do not fall within an assigned Canadian MRSA epidemic type but is associated with multi-locus sequence types 12 and 13.

**Fig. 2:**
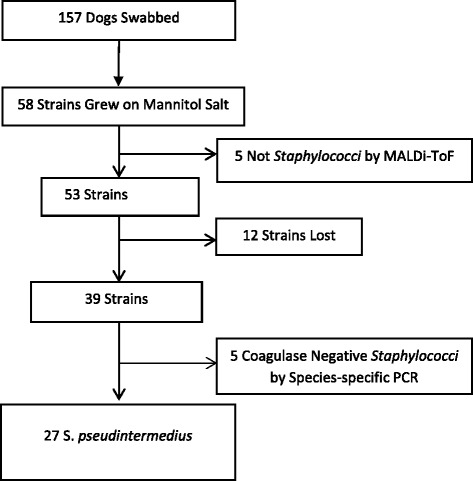
Recruitment of Dogs in Two Aboriginal Communities, and Identification of Staphylococcal Isolated

One hundred fifty-seven dogs were sampled in two communities, including 57 from the community in which humans were surveyed, and 100 from another Aboriginal community in Labrador (Fig. 2). 34 strains of coagulase positive Staphylococci were identified, including 27 *S. pseudintermedius*, 8 *S. aureus* and 1 *S. schleiferi*. The *S. aureus* were all methicillin-susceptible and were typed as the following: t002 (37.5 %, 3/8), t008, t012, t037, t038, and t14525. The methicillin-susceptible *S. aureus* colonization rate in dogs was 8/157 (5.1 %, 95 % C.I. = 1.7–8.5 %). There were no MRSA detected in dogs.

## Discussion

Our study demonstrated a high rate of CA-MRSA colonization among humans in an Aboriginal community. Colonization was 87.1 % identical spa type, suggesting predominantly person to person spread of a single organism. The typing differs from CA-MRSA clinical isolates in Alberta [8], which are more diverse, and from the CA-MRSA clinical isolates in Northern Saskatchewan, which are more CAMRSA7 [16]. The Aboriginal population studied is isolated with limited intermixing with surrounding populations.

Traditional risk factors did not explain colonization well, but crowding and recreational dog exposure remained significant in a multivariate model. Other variables considering dog exposure (dog in the house, own a dog, feed a dog) were not predictive, suggesting that recreational dog exposure alone may be significant by chance. Recreational dog use was somewhat broadly defined, and may have included varying levels of dog exposure. Overall the epidemiological analysis did not suggest that dog-human transmission of MRSA was common. Typing results demonstrated rare dog colonization with human strains (only 8/157 dogs carried *S. aureus*, 5.1 %) and rare human colonization with Staphylococci other than *S.aureus,* which could have been from animal origin (3/111, 2.7 %). This would suggest a very low level of interspecies Staphylococcal transmission in this setting. The observed level of human and dog interaction was moderate to low, with many dogs being fed by several different humans and sleeping outside houses, in a more feral lifestyle.

Rooms in the house, an indirect measure of human crowding, was a significantly negative predictor of MRSA colonization. This result agrees with previous suggestions that human to human distance is relevant. If colonization is transmitted by skin to skin contact, then crowded living conditions may increase this contact and promote transmission. One considered variable indirectly measuring personal hygiene (number of sinks in house) was not significant, although additional hygiene factors were not explored.

Previous literature has examined human MRSA colonization rates in community settings. A meta-analysis of eighteen studies found that only three studies (*N* = 4452) performed surveillance outside of healthcare settings [12]. Among these three studies, CA-MRSA colonization was 0.76 %. These three populations did not represent remote Aboriginal communities. Screening of remote Aboriginal communities in Australia for MRSA colonization revealed 42 % colonization in one community (39 % identical strains) and 24 % in a second community (17 % identical strains) [29]. This would suggest a large difference in community colonization prevalence between Aboriginal and non-Aboriginal communities. In that Aboriginal communities may be geographically and culturally isolated from non-Aboriginal communities, MRSA epidemiology may be unique in these settings. Unknown or novel risk factors may contribute to high colonization prevalence.

Our results suggest that traditional risk factors including healthcare exposure, correctional exposure, daycare exposure, contact with someone with MRSA infection, tattoos or piercings, or access to sinks are not relevant risk factors in this community. The low statistical performance of our multivariate predictive model suggests that unmeasured risk factors contribute more than measured risk factors to MRSA colonization. We have not directly measured animal exposure among humans, relying instead on self-report, however our molecular analysis does not suggest that interspecies transmission is occurring in this setting.

Among patients hospitalized with MRSA infection, Aboriginals have different risk factors than non-Aboriginals. Aboriginals are more likely to be younger, more likely to have skin and soft tissue infection, and are more likely to have acquired infection in the community [11]. Also MRSA strains are genetically different [11]. Based on high colonization prevalence and the absence of traditional risk factors, MRSA control among Aboriginal populations may have to be considered differently than among other populations.

Strengths of our study include population-based surveillance undertaken well away from the healthcare setting. This approach may provide more accurate colonization prevalence than hospital-based testing. Limitations of our study include approximately 40 % sampling representation of the village population, which may have influenced our colonization prevalence estimate, through sampling bias. The nasal method of swab collection may have missed additional colonization present on other body sites. Twelve animal strains were lost from analysis, which may have influenced our interpretation. Not all dogs sampled were living in the same village where humans were sampled. Transportation time for swabs between collection and testing may have been up to five days, which may have allowed death of organisms in transit. We did not account for household clustering of risk factors.

Further research to determine unmeasured risk factors may be helpful in designing interventions to reduce MRSA infection rate. Risk factors such as crowding are difficult to influence, but the beneficial impact of education in Saskatchewan suggests that behavioral risk factors may be present and amenable to change. Although antibiotic use was not a significant predictor in our study, this risk factor has been shown to be associated with MRSA colonization in other populations [30], and may be amenable to intervention. We have no evidence to suggest that intervening to reduce dog-human contact would influence human MRSA colonization. 

## Conclusions

Our study demonstrated a high rate of MRSA colonization among humans and no evidence for human to animal MRSA transmission.

## Additional file

Additional file 1: Appendix Risk Factor Questionnaire

## Abbreviations

CA-MRSA: Community-acquired Methicillin-resistant *Staphylococcus aureus*
MALDi-ToF: Matrix-assisted laser desorption/ionization-time of flight
MRSA: Methicillin-resistant *Staphylococcus aureus*
OR: Odds ratio
PCR: Polymerase chain reaction
