# Supplementary material for: A cross sectional study of animal and human colonization with Methicillin-Resistant Staphylococcus aureus (MRSA) in an Aboriginal community
Source: BMC Public Health. 2016 Jul 19;16:595. doi: 10.1186/s12889-016-3220-9 (PMC4950257; doi:10.1186/s12889-016-3220-9)
Supplement: Additional file 1: — Appendix Risk Factor Questionnaire [file 12889_2016_3220_MOESM1_ESM.docx]

Appendix Risk Factor Questionnaire

**Human Survey Questionnaire**

**Section One: Case Information**

*Note to interviewer: Some questions below may need to be revisited more than once during the interview to ensure responses are complete (e.g. source of information used). Please write legibly. Complete one questionnaire for each person (adult or child).*

***Script****: To help us better understand you, I will be asking you questions about you and your family and your house. You only need to answer questions that you feel comfortable with.*

**Date of Interview (mmm/dd/yyyy):_________**

**Name of interviewer: __________________________**

**1.1 Unique Patient Identifier: _________________**

**1.3** **Written consent obtained?**

Yes

No (if no, terminate interview)

**1.4 Demographic Information**

**1.4.1** **Date of Birth DD/MM/YY ________________**

**1.4.2** **Gender**

⁯ Male

⁯ Female

⁯ Other

**1.4.3** Location of residence: (mark on satellite image of village) Address________________________

a) How many rooms are there in your household? _________

Rooms Include kitchen, bedrooms, finished rooms in attic or basement, etc.

Do not count bathrooms, halls, vestibules and rooms used solely for business purposes.

b) How many people sleep in your house?

_______ adults over 18 years

_______children under 18 years

c) How many sinks are in your house?_____________

d) What is the water source?

□ carried from outside the house

□ piped into the house

□ from well

□ from surface water source

e) What toilet is in the house?

□ none

□ outside outhouse

□ inside toilet to septic tank

□ inside toilet to community sewage

**1.4.4 Ethnicity:**

⁯ Aboriginal (mark specific category)

⁯ Innu

⁯Inuit

⁯ Mixed-eg indicates multi ethnic background. Please specify________________

⁯ Caucasian _______

Other, please specify______

⁯ Unknown/not available________

**Section Two: Study Investigation**

**2.1 Date of specimen collection:** _____/____/____ (MM/DD/YY) ⁯ Missing

**2.2** **Date of specimen receipt in St. John’s**: _______/_____/_______(MM/DD/YY) ⁯Missing

**2.3 Site of specimen:** ⁯ Nasal

⁯ Other (specify) ____________

**Section Three: Past Medical History and Risk Factors**

***Script****: To help us better understand the risk factors for MRSA infection, I will be asking you questions about your past medical history, interactions with the healthcare system, living arrangements, social networks, and relevant activities .*

***Note to interviewer:*** *The following information can be extracted from laboratory reports, interview or chart review (if indicated)*

**CONTACT WITH THE HEALTHCARE SYSTEM AND MEDICAL HISTORY:**

- 1. Within the past 12 months, have you had any of the following:
     1. **Admission to hospital OR nursing home OR long term care facility**

**(excluding ER visits with no admission)**

□ Yes □ No □ Don’t Know □ Refused

- - - 1. **If yes, describe your stay:**

□ <48 hours □ 48 hours or more

□ ICU □ ward

- - 1. **Surgery?**

□ Yes □ No □ Don’t Know □ Refused

- - 1. **Indwelling medical devices (catheter, IV line, etc)?**

□ Yes □ No □ Don’t Know □ Refused

- - 1. **Have you been on dialysis?**

□ Yes □ No □ Don’t Know □ Refused

- - 1. **History of MRSA infection or colonization?**

□ Yes □ No □ Don’t Know □ Refused

3.1.5.1 If yes, please indicate the site of specimen collection:

□ Skin

□ Deep tissue

□ Blood

□ Sputum/respiratory

3.1.5.2. If yes, please indicate the date of specimen collection (MM/DD/YY)_______

- - 1. **Antibiotic use?**

□ Yes □ No □ Don’t Know □ Refused

- - - 1. **If yes, how many courses of antibiotics did you have?**

□ Don’t Know □ Refused □ Number of courses of antibiotics________

- 1. **Within the past 12 months have you had:**

- - 1. A skin infection?

□ Yes □ No □ Don’t Know □ Refused

**3.2.1.1. If yes**, was it:

□ ⁯4 or more weeks ago ⁯ □ less than 4 weeks ago?

**3.2.1.2 If more than 4 weeks ago,** was it cleared to your satisfaction?

□ Yes □ No □ Don’t Know □ Refused

If not recovered, please specify_______________________________________________________

3.2.1.2 Did a doctor cut open your infection site with a scalpel or other instrument to drain the site?

□ Yes □ No □ Don’t Know □ Refused

3.2.1.3 Were you taken to the operating room for MRSA?

□ Yes □ No □ Don’t Know □ Refused

**3.2.2 A non-skin MRSA infection?**

⁯Pneumonia (lung)

⁯Bacteraemia (bloodstream infection)

⁯Urinary Tract Infection (bladder)

⁯Meningitis

⁯Other (*specify*) ______________________

**3.2.2** Contact with someone else with a (similar) skin infection?

□ Yes □ No □ Don’t Know □ Refused

**3.2.3.** Contact with someone with MRSA?

□ Yes □ No □ Don’t Know □ Refused

3.2.4. Lived with someone with MRSA?

□ Yes □ No □ Don’t Know □ Refused

3.2.5. Did you use injection drugs?

□ Yes □ No □ Don’t Know □ Refused

3.2.6. Have a new tattoo or piercing?

□ Yes □ No □ Don’t Know □ Refused

**3.3 Have you had a chronic skin condition within the past 12 months (not MRSA)?**

□ Yes □ No □ Don’t Know □ Refused

**3.3.1 If yes, Please specify:___________**

**LIVING ARRANGEMENTS:**

**3.7 Within the past 12 months, have you been exposed to the following settings:**

3.7.1 Healthcare / LTC facility? (not admitted)

□ Yes □ No □ Don’t Know □ Refused

**3.7.1.1 If yes, were you**

⁯□ employee

⁯□ inpatient / resident

⁯□ volunteer

□ Visiting an inpatient

3.7.2. Correctional facility?

□ Yes □ No □ Don’t Know □ Refused

**3.7.2.1 If yes, were you**

⁯□ worker

□ ⁯resident

3.7.3. Daycare center?

□ Yes □ No □ Don’t Know □ Refused

**3.7.3.1 If yes, were you**

⁯□ worker

□ ⁯attendee

□ parent of child in daycare

3.7.4 Homeless shelter / group home?

□ Yes □ No □ Don’t Know □ Refused

**3.7.4.1. If yes, were you**

⁯□ worker

⁯□ resident

⁯□ volunteer

3.7.5. Veterinary / animal worker?

□ Yes □ No □ Don’t Know □ Refused

**3.8 Are you a household member or close contact of a person exposed to the following settings?”**

**3.8.1** Healthcare / LTC facility?

□ Yes □ No □ Don’t Know □ Refused

**3.8.2** Correctional facility?

□ Yes □ No □ Don’t Know □ Refused

**3.8.3** Daycare center?

□ Yes □ No □ Don’t Know □ Refused

**3.8.4** Homeless shelter/group home

□ Yes □ No □ Don’t Know □ Refused

**3.8.5** Veterinary / animal worker?

□ Yes □ No □ Don’t Know □ Refused

**Section Four: Dog Exposure**

4.1 Does a dog sleep inside your house?

□ Yes How many dogs? _________

□ No

4.2. Do you own a dog?

□ Yes

□ No

4.3. Do you feed a dog regularly?

□ Yes

□ No

4.4 Do you use dogs for your work or recreation?

□ Yes Explain (hunting, transportation, other use) ____________

□ No

4.5 Do you have contact with a dog with an open wound?

□ Yes

□ No

Script: *Thank you for your assistance with our study.*
